# Symptomatic cervical disc herniation in teenagers: two case reports

**DOI:** 10.1186/1752-1947-7-42

**Published:** 2013-02-12

**Authors:** Toshiki Abe, Naohisa Miyakoshi, Michio Hongo, Takashi Kobayashi, Tetsuya Suzuki, Eiji Abe, Yoichi Shimada

**Affiliations:** 1 Department of Orthopedic Surgery, Akita Kumiai General Hospital, 1-1-1 Iijima-Nishifukuro, Akita, 011-0948, Japan; 2 Department of Orthopedic Surgery, Akita University Graduate School of Medicine, 1-1-1 Hondo, Akita, 010-8543, Japan

## Abstract

**Introduction:**

The development of a symptomatic herniated cervical disc before the age of 20 is extremely rare. Sporadically reported cases of patients with cervical disc herniation under the age of 20 usually have had underlying disease.

**Case presentation:**

Case 1: A 19-year-old Asian man visited our clinic and presented with progressive pain in his upper left scapula and weakness of the left deltoid and biceps brachii muscles. C5 radiculopathy by soft disc herniation at C4-C5 without calcification was diagnosed. Microsurgical posterior foraminotomy was performed and he recovered completely eight weeks after the surgery.

Case 2: A 15-year-old Asian man presented with difficulty in lifting his arm and neck pain on the right side. Neurological examination showed weakness of the right deltoid and biceps brachii muscles. A magnetic resonance imaging scan demonstrated a herniated intervertebral disc in the right C4-C5 foramen. The patient was treated conservatively and put under observation only, and had completely recovered eight weeks after admission.

**Conclusion:**

Although extremely rare, symptomatic cervical disc herniations may occur even in the younger population under the age of 20 without any trauma or underlying disease. Favorable outcomes can be achieved by conventional treatments for cervical disc herniation.

## Introduction

Cervical disc herniation is generally caused by degeneration of the cervical vertebrae. Symptomatic cervical disc herniation is a common cause of radiculopathy, and there is a clear peak incidence in the fourth and fifth decades [1]. Because disc degeneration advances with age, the development of a symptomatic herniated cervical disc before the age of 20 is extremely rare. All of the sporadically reported cases with cervical disc herniation before the age of 20 had underlying disease, such as Klippel-Feil syndrome (KFS) [2]. To the best of our knowledge, symptomatic cervical disc herniation occurring in teenaged patients without underlying disease has not been previously reported in the English literature. We report herein two cases of symptomatic cervical disc herniation with no trauma or underlying disease in teenaged patients.

## Case presentations

### Case 1

A 19-year-old Asian man was experiencing pain in his upper left scapula. One week after the onset of the pain, he had difficulty lifting his left arm and visited our clinic. The pain radiated from the left side of his neck through the scapular region with neck extension, and was controlled with analgesic medication. He had no relevant family history, such as congenital spine abnormalities, and had no past history of trauma or birth injuries. Neurological examination showed weakness of the left deltoid and biceps brachii muscles (power, 2 out of 5 and 4 out of 5, respectively), decreased sensation of his left lateral upper arm, and a hypotonic left biceps tendon reflex. The Spurling test, which is positive if the patient produces symptoms when the patient laterally flexes and extends the neck with the examiner applying axial pressure on the spine, was positive on the left side. Cervical radiculopathy score, an assessment system proposed by Tanaka evaluating pain, disability, and neurological status on a 20-point scale, with 20 being no pain, disability, and neurological findings, was 5 out of 20 [3].

**Figure 1:**
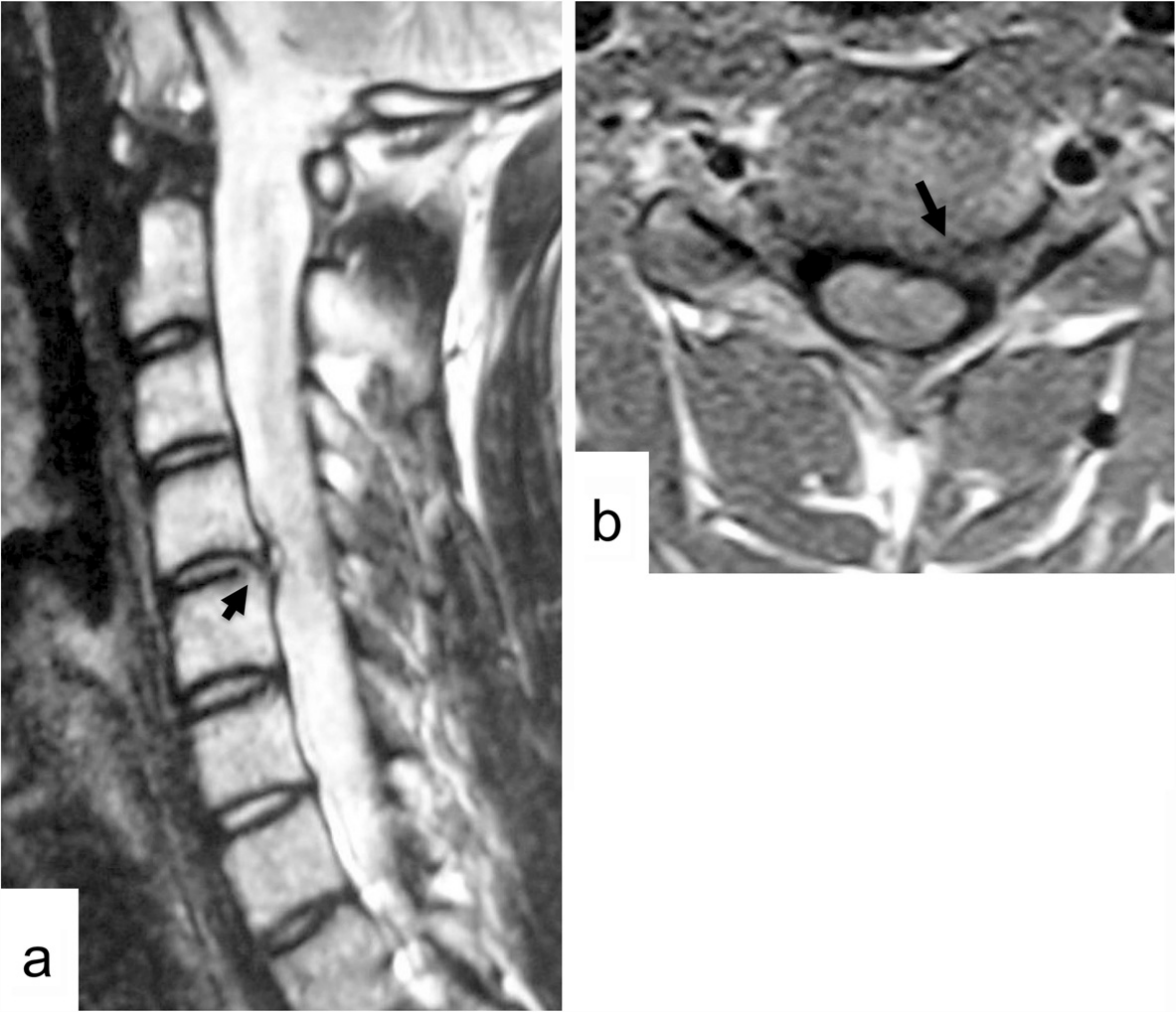
**Case 1.** Left parasagittal T2-weighted magnetic resonance imaging (MRI) scan of the cervical spine **(a)** and axial T1-weighted MRI scan at the C4-C5 level **(b)** showing a herniated intervertebral disc in the left C4-C5 intervertebral foramen (arrows).

Plain radiography was normal with no evidence of degeneration or calcification. Magnetic resonance imaging (MRI) demonstrated a herniated intervertebral disc in the left C4-C5 intervertebral foramen (Figure 1a, b). C5 radiculopathy by soft disc herniation at C4-C5 was diagnosed.

Initial management consisted of pharmacologic pain control, relative rest, and a hard cervical collar worn in a position to maximize arm pain reduction. Mechanical traction was not applied. However, since there were no signs of improvement in muscle weakness despite conservative treatment for two weeks following admission, microsurgical posterior foraminotomy was performed [4]. The patient’s pain and motor strength improved gradually after surgery. The patient had recovered completely eight weeks after the surgery. Cervical radiculopathy score was 19 at the final follow-up three years after surgery.

### Case 2

**Figure 2:**
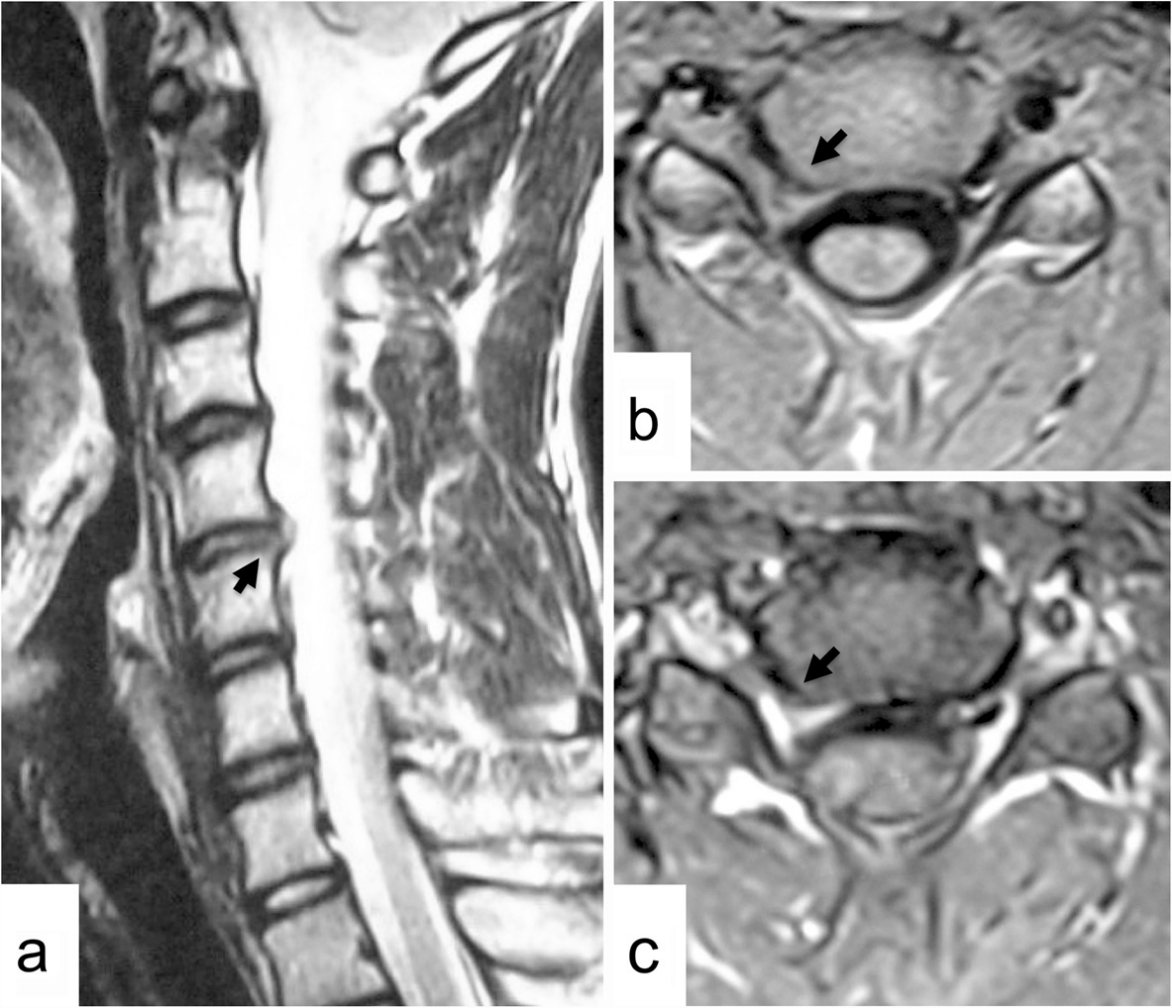
**Case 2.** Right parasagittal T2-weighted magnetic resonance imaging (MRI) scan of the cervical spine **(a)** and axial T1-weighted MRI scan at the C4-C5 level **(b)** showing a herniated intervertebral disc in the right C4-C5 intervertebral foramen (arrows). The herniated disc is partially enhanced after contrast medium **(c)** (arrow).

A 15-year-old Asian man experienced sudden neck pain on the right side and had difficulty lifting his right arm when he woke up one morning. The symptoms continued for two weeks and he presented to our clinic. His neck pain increased with neck extension. He had no relevant family or past history that included congenital abnormality or trauma. Neurological examination showed weakness of the right deltoid and biceps brachii muscles (power, 2 out of 5 and 4 out of 5, respectively), and a hypotonic right biceps tendon reflex. No obvious sensory loss was present. Cervical radiculopathy score was 13 out of 20. Plain radiography showed no abnormality. An MRI scan demonstrated a herniated intervertebral disc in the right C4-C5 intervertebral foramen (Figure 2a, b). The herniated disc showed partial contrast enhancement after administration of Gd-diethylenetriaminepentaacetic acid (Gd-DTPA) (Figure 2c).

Since the muscle weakness improved within the first week after admission, the patient was treated conservatively and put under observation only. Muscle strength had recovered to 4 by the third week, and after eight weeks, the patient had completely recovered. Cervical radiculopathy score was 20 at the final follow-up of two years.

## Discussion

Cervical disc herniation, a well-known cause of cervical myelopathy and radiculopathy, is more common in men and often occurs in those 40 to 60 years of age, with an average age of around 50 years. C5-C6 is frequently involved in the case of cervical myelopathy, whereas C6-C7 is more common in radiculopathy [5]. In both cases presented, C4-C5 was the affected level, with deltoid muscle strength of 2 and difficulty in arm lifting (using the positive drop-arm sign). Since C4-C5 is the level with the greatest rotation and lateral flexion movement among the middle and lower cervical vertebrae [6], and since mobility of the cervical vertebrae tends to decrease with advancing age [7], it is possible that this level was affected due to the tendency for C4-C5 to be vulnerable to loading in young people with no degeneration.

There have been scattered reports of cervical disc herniation occurring in young people [2,8,9]. However, all of the previously reported cases had underlying disease, that is, a complication of intervertebral disc calcification or KFS [2]. Samartzis *et al*. [2] reported a 16-year-old KFS boy with occipitalization of C1 and fusion of C2-C3, and C4-T1 showed cervical disc herniation at his hypermobile segment of C3-C4. In an extensive search of the literature, there were no reports of symptomatic cervical disc herniation occurring in teenaged patients without underlying disease. In this regard, the two cases presented here are extremely rare.

In general, cervical disc herniation responds well to conservative treatment. Saal *et al*. reported that herniated cervical disc with radiculopathy was successfully managed in 93% of the patients with nonoperative management, including relative rest, cervical collar, analgesic medicine, traction, and physical training [10]. The authors described that no patients had progressive neurological loss or reached a neurological catastrophe. However, the timing of the surgical intervention for the patient with motor deficit is still unclear. The patient in Case 1 underwent surgical decompression because there were no signs of improvement in motor and sensory function after two weeks of conservative treatment. Posterior decompression was selected rather than ventral decompression and fusion. The advantages of foraminotomy are to preserve the motion segment and to minimize the future risk of adjacent segment disorder for this younger age. Another advantage is to reduce the period of postoperative management in contrast with the latter procedure.

Cervical disc herniation is known to resolve spontaneously in the same way as lumbar disc herniation [11]. In Case 2, the herniation was enhanced on an MRI scan after Gd-DTPA administration, indicating that spontaneous resolution could be anticipated, and muscle strength improved within a week after admission. Therefore, surgery was not indicated, and a favorable outcome was obtained with conservative treatment.

In both cases, a favorable outcome was achieved by conventional treatments for cervical disc herniation, but differences in pathology compared with the common middle-aged onset of this disorder remain a question for future investigation.

## Conclusion

Although extremely rare, symptomatic cervical disc herniations may occur even in the younger population under the age of 20 without any trauma or underlying disease. Favorable outcomes can be achieved by conventional treatments for cervical disc herniation.

## Consent

Case 1: Written informed consent was obtained from the patient for publication of this case report and any accompanying images. A copy of the written consent is available for review by the Editor-in-Chief of this journal.

Case 2: Written informed consent was obtained from the patient’s legal guardian for publication of this case report and any accompanying images. A copy of the written consent is available for review by the Editor-in-Chief of this journal.

## Competing interests

The authors declare that they have no competing interests.

## Authors’ contributions

Surgery was performed by TA, TS, and EA. TA, NM, and EA were the major contributors in writing the manuscript. MH and TK analyzed and interpreted the patient data. EA and YS supervised the whole work. All authors read and approved the final manuscript.
